# Membrane Bioreactor for Simultaneous Synthesis and Fractionation of Oligosaccharides

**DOI:** 10.3390/membranes12020171

**Published:** 2022-01-31

**Authors:** Vanessa A. Botelho, Marília Mateus, José C. C. Petrus, Maria Norberta de Pinho

**Affiliations:** 1 Institute of Food Engineering, Universidade Federal do Pará, R. Augusto Corrêa, 01-Guamá, Belém 66075-110, PA, Brazil; vanessalbres@ufpa.br; 2 Institute for Bioengineering and Biosciences (iBB) and Department of Bioengineering, Instituto Superior Técnico, Universidade de Lisboa, Av. Rovisco Pais 1, 1049-001 Lisboa, Portugal; 3 Department of Chemical and Food Engineering, Centro Tecnológico (CTC), Universidade Federal de Santa Catarina, Caixa Postal 476, Florianópolis 88040-970, SC, Brazil; 4 Centre of Physics and Engineering of Advanced Materials (CeFEMA) and Department of Chemical Engineering, Instituto Superior Técnico, Universidade de Lisboa, Av. Rovisco Pais 1, 1049-001 Lisboa, Portugal; marianpinho@tecnico.ulisboa.pt; 5 Associate Laboratory of Physics for Materials and Emergent Technologies (LaPMET), Instituto Superior Técnico, Universidade de Lisboa, 1049-001 Lisboa, Portugal

**Keywords:** membrane bioreactor, membrane filtration, oligosaccharides fractionation, oligosaccharides synthesis

## Abstract

Galacto-oligosaccharides (GOS) are prebiotic sugars obtained enzymatically from lactose and used in food industry due to their nutritional advantages or technological properties. Selective mass transport and enzymatic synthesis were integrated and followed using a membrane bioreactor, so that selective removal of reaction products may lead to increased conversions of product-inhibited or thermodynamically unfavorable reactions. GOS syntheses were conducted on lactose solutions (150 g·L^−1^) at 40 °C and 10 U_β-galactosidase_.mL^−1^, and sugar fractionation was performed by cellulose acetate membranes. Effects of pressure (20; 24 bar) and crossflow velocity (1.7; 2.0; 2.4 m·s^−1^) on bioreactor performance were studied. Simultaneous GOS synthesis and production fractionation increased GOS production by 60%, in comparison to the same reactions promoted without permeation. The presence of a high-molecular-weight solute, the enzyme, in association with high total sugar concentration, leads to complex selective mass transfer characteristics. Without the enzyme, the membrane presented tight ultrafiltration characteristics, permeating mono- and disaccharides and retaining just 25% of trisaccharides. During simultaneous synthesis and fractionation, GOS-3 were totally retained, and GOS-2 and monosaccharides were retained at 80% and 40%, respectively. GOS synthesis—hydrolysis evolution was strongly dependent on crossflow velocity at 20 bar but became fairly independent at 24 bar.

## 1. Introduction

Galacto-oligosaccharides (GOS) are prebiotic sugars that can be used in the food industry for their nutritional advantages or technological properties. They can be obtained enzymatically from lactose. Cheese whey is an inexpensive source of lactose and could be an option for substrates, with the additional advantage of reducing the pollutant charge of dairy wastes.

β-galactosidase is a transferase enzyme; when it transfers the galactosyl moieties of lactose to water molecules, two monosaccharides are released (hydrolysis reaction) and, when the transfer occurs to another saccharide molecule unit, GOS are formed (synthesis reaction) [1]. In dilute aqueous solutions the equilibrium is shifted to favor hydrolysis over synthesis, as is highlighted by Botelho-Cunha et al. (2010) [2], and, consequently, it is necessary to increase the lactose concentration to decrease water molecule availability [3] and increase the GOS production. The available enzymes and their characteristics, reaction and purification conditions, outcomes and main industrial companies have been reviewed by Vera et al. (2016) [4].

Membranes have been used not only for fractionation of GOS reaction mixtures [2] or model solutions of monosaccharides [5,6] but also to retain the enzyme during continuous GOS synthesis [7] or to purify commercial enzyme-free GOS mixtures [8,9]. The continuous enzymatic production of GOS by Cao et al. (2020) [7] was conducted and studied in a 10 kDa pilot-scale well-instrumented/monitored membrane bioreactor (EMB). Under the biochemical reaction conditions (~95 U_β-galactosidase_.g^−1^, 30% (*w*/*w*) lactose feed, 50 °C, 1.8 h residence time), the GOS products were mainly disaccharides but also reached oligomers of 3–6 monosaccharide units. The continuous enzyme membrane bioreactor (EMB) permeate composition of the runs showed steady (for 120 h) and reproducible (two runs) behaviors at pressures of 0.3–0.8 bar, which demonstrated remarkable enzyme operation stability in the EMR system.

Membrane filtration has also been used for processing other enzymatically produced oligosaccharide mixtures and was revised by Su et al. (2020) [10], who stressed the urgency of studying the implications of coupling reaction and membrane filtration steps not considered by previous works. A few more recent works addressed the subject and should be mentioned, namely for a cascade of ultrafiltration (UF) and nanofiltration (NF) membranes inserted in a stirred cell used in a preliminary study on the viability of producing xylo-oligosaccharide fractions as feeds for prebiotic bacteria fermentations [11]. Interactions between simultaneous enzymatic reaction and UF using a multi-channel ceramic 1-kDa membrane were investigated during the production of fructo-oligosaccharides at high substrate concentrations (60% sucrose) [12]. Although no shear effects due to crossflow velocity were looked after, pressure effects on the free-flowing fructosyltransferase in operation of the membrane bioreactor were investigated, showing that this particular enzyme lost ~15% of its hydrolysis activity after circulation at 0.5 m·s^−1^ for 2 h under transmembrane pressure of 2.5 bar. This is relevant information, though the most important reaction is the synthesis and not just that of substrate hydrolysis (measured in standard conditions, outside the reactor).

In the present work batch GOS synthesis reactions with soluble enzymes are processed simultaneously with ultrafiltration membranes that under operation conditions display higher GOS retention than those presented in [7], and so our study addresses a very complex multi-component mixture covering a very broad range of molecular weights. The retention of enzymes is assured, and the fractionation of intermediate/low-molecular-weight reaction products is investigated.

Emphasis has been given to the combination of selective mass transport with chemical reactions, so that the selective removal of reaction products would lead to the increase in the conversion in reactions that are product-inhibited or thermodynamically unfavorable [3]. The main objective of this work arises in this framework, as the integration of enzymatic oligosaccharide synthesis and membrane filtration operating conditions is designed in an integrated form, to investigate pressure and shear effects on batch GOS production under filtration.

## 2. Materials and Methods

### 2.1. Biological and Chemical Materials

A commercial preparation of **β**-galactosidase from *Kluyveromyces lactis*, Lactozym 3000 L HP-G (Novo Nordisk A/S, Bagvaerd, Denmark), was used. The chemicals used to prepare the reaction media and standard solutions were lactose monohydrate and glucose anhydrous from Merck (Darmstadt, Germany); galactose, melezitose and bovine serum albumin (BSA), fraction V, from Sigma (St. Louis, MI, USA) and raffinose from Panreac (Chicago, IL, USA). Cellulose acetate with 38.9% acetyl content was purchased from Sigma. The other chemicals used were all of analytical grade. Deionized water was used for preparation of media and membrane-rinsing solutions, while for chromatographic standards, Milli-Q ultrapure water (through Q-POD unit) was used.

### 2.2. Enzyme Assays

#### 2.2.1. Enzyme Activity

The enzyme activity was determined following the initial minutes of a batch hydrolysis reaction of 30 mM lactose solution, pH 6.5, at 25 °C, prepared with 50 mM sodium and potassium phosphate buffer containing 10 mM MgCl_2_ [13]. The commercial enzyme formulation (0.5 g) was added to a lactose solution (10 mL), and samples were collected along the reaction time. To stop the reaction, the samples collected (750 μL) were immediately added into tubes containing the same amount of trichloroacetic acid, 0.05 N. The amount of glucose released was quantified by HPLC, under the conditions explained in Section 2.3. One unit of enzyme activity was defined as the amount of enzyme activity catalyzing the release of 1 μmol glucose per minute, under the defined conditions.

#### 2.2.2. Total Protein Concentration

Bradford’s method was used to quantify the decrease in protein amount inside the reactor. BSA solutions were prepared with 50 mM sodium and potassium phosphate buffer containing 10 mM MgCl_2_ and used as standards in two different calibration curves: standard (0.1–1.0 g·L^−^^1^) and micro-method (0.01–0.1 g·L^−^^1^). In the standard method, 100 μL of sample or standard solution was mixed with 5 mL of Bradford reagent and, in the micro-method, the sample- or standard-to-reagent ratio was 300 μL to 3 mL. Absorbances were measured at 540 nm after 5 min (Shimadzu UV-1700, Shimadzu, Kyoto, Japan).

### 2.3. Sugar Analysis

**Figure 1:**
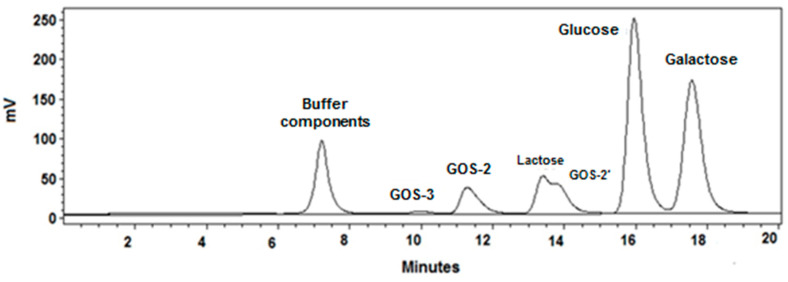
HPLC chromatogram of a sample of the reaction medium (150 g·L^−^^1^ lactose, 10 U·mL^−^^1^ β-galactosidase, 40 °C and pH 6.5) at 40 min of batch conversion.

Quantitative analyses of all carbohydrates were carried out by high-performance liquid chromatography (HPLC). The system was equipped with a Waters 600 Controller, Waters 717 plus auto sampler and Gilson 133 refractive index detector. The saccharides’ separation was performed at 80 °C with Supelcogel™ Ag column (Supelco Inc., Bellefonte, PA, USA). Elution was isocratic at 0.5 mL·min^−^^1^ of pure water continuously sparged (15 mL·min^−^^1^) with helium. The standards used for chromatographic peak area calibration were: glucose, lactose and melezitose, respectively mono-, di- and trisaccharide. All concentrations of disaccharides different from lactose, named GOS-2 and GOS-2′, were assessed with lactose calibration and those of tri- and tetrasaccharides, named GOS-3 and GOS-4, respectively, were assessed with melezitose calibration. The total GOS produced in each reaction was the sum of all detected and quantified fractions of oligosaccharides. Figure 1 shows an example of the chromatograms obtained during a GOS synthesis reaction.

### 2.4. Membrane Bioreactor

#### 2.4.1. Experimental Set-Up

**Figure 2:**
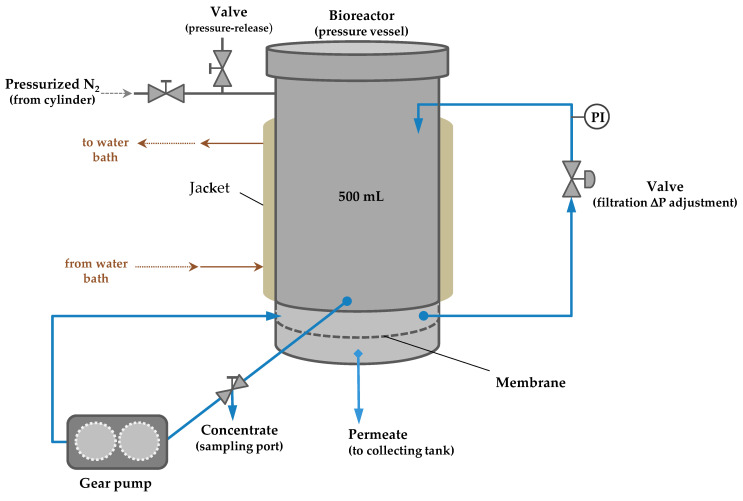
Process scheme of the P-28 Celfa unit for simultaneous enzymatic synthesis and membrane filtration.

The P-28 Celfa unit, used in this work, integrates a reaction vessel of 500 mL with a membrane filtration cell of an effective membrane surface area of 26.4 cm^2^ (Figure 2). The feed reaction mixture circulates tangentially over the active layer surface of the membrane, and the fraction permeating through the membrane is collected in a permeate channel. The fraction that does not permeate (retentate) returns to the feed tank and can be sampled through a valve located in the bottom of the feed container, above the membrane. One gear pump with velocity control makes the fluid circulation. The system was pressurized by nitrogen injection. Samples from permeate and retentate (1 mL) were analyzed by HPLC. From the retentate samples, 750 μL were immediately taken and mixed with the same amount of 0.05 N trichloroacetic acid solution to stop the enzyme reaction.

#### 2.4.2. Membranes

Cellulose acetate (CA) membranes were prepared by phase-inversion method. The casting solutions were prepared with cellulose acetate (17%), acetone (56%) and formamide (27%). The membranes were cast at room temperature and, after 30 s of evaporation time, were immersed in deionised water (0–3 °C) for a gelation process of approximately 2 h. The membranes formed were kept at 5 °C in 15% *v/v* ethanol solution.

A new membrane was used for each experiment. Prior to use, the membranes were subjected to a compaction pre-treatment for 2 h with pure water recirculation at 30 bar. The volumetric permeate flux of water at 25 °C (*J_v_*) was measured for all membranes at different pressures.

#### 2.4.3. Process Operation Conditions

The GOS syntheses were carried out at 40 °C with 400 mL of buffered solution at 150 g·L^−^^1^ lactose and 10 U·mL^−^^1^ β-galactosidase. After the desired temperature was reached, and the volumetric permeate flux of water (*J_v_*) and crossflow velocity were stabilized (1.7; 2.0 or 2.4 m·s^−^^1^), the enzyme was added, the cell immediately closed, and nitrogen was supplied to reach the pre-established pressure (20 or 24 bar). The permeate flux from the saccharide reaction mixture was measured, and samples from permeate and retentate fractions were taken along the reaction time to quantify the number of saccharides (as described in Section 2.3) to calculate the observed rejection, *R_obs_*.

### 2.5. Membrane Regeneration

The membrane resistance, *R_m_*, was calculated by measuring its hydraulic permeability, *L_p_*, at 40 °C:

(1) $$L_{p}=\frac{1}{\mu \times R_{m}}$$

where *μ* is the water viscosity. The reaction mixture (initially 150 g·L^−^^1^ lactose and 10 U·mL^−^^1^ β-galactosidase, in buffered solution) was converted in the membrane reactor, under filtration conditions, for 60 min at 20 bar and 40 °C, and the total resistance to permeation, *R_t_*, was calculated by

(2) $$J_{rm}=\frac{\Delta P}{\mu \times R_{t}}$$

where *J_rm_* is the reaction mixture permeation flux, and *R_t_* is the sum of *R_m_*, *R_cp_*, *R_rf_* and *R_if_*, respectively, the contributions of the membrane, of the concentration polarization layer, and of the reversible and irreversible fouling. After the conversion/separation process, the reactor was rinsed with water circulation for 15 min to remove any reversible fouling. The water permeate flux was measured again (*J_w′_*) under the same experimental conditions:

(3) $$J_{w\prime }=\frac{\Delta P}{\mu \times \left(R_{m}+R_{if}\right)}$$

and *R_if_* could be calculated. Thereafter, the reactor was emptied, cleaned with 0.01 M NaOH alkaline solution for 15 min and rinsed with water circulation. The water permeate flux was measured again (*J_w″_*), and *R_m′_*, the resistance of the washed membrane, could be calculated through Equation (4).

(4) $$J_{w\prime \prime }=\frac{\Delta P}{\mu \times R_{m\prime }}$$

## 3. Results

### 3.1. Membrane Bioreactor Characterization and Optimization

**Figure 3:**
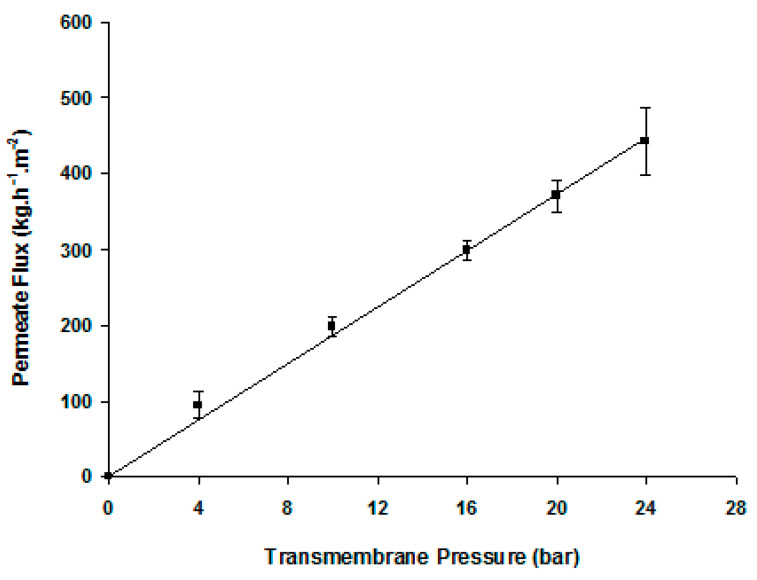
Average hydraulic permeability of the cellulose acetate tight ultrafiltration membranes at 25 °C, *L_p_* = 15.3 ± 3.1 Kg·h^−1^·m^−2^·bar^−1^ (out of 9 different membranes).

Figure 3 displays the straight line of the variation of the pure water permeate flux as a function of the transmembrane pressure. The slope of this line is the CA membrane hydraulic permeability, *L_p_* = 15.5 ± 3.1 kg·h^−1^·m^−2^·bar^−1^. Table 1 displays the membrane retention coefficients to a set of reference solutes. The membrane practically does not retain mono- or disaccharides and slightly retains trisaccharides.

**Figure 4:**
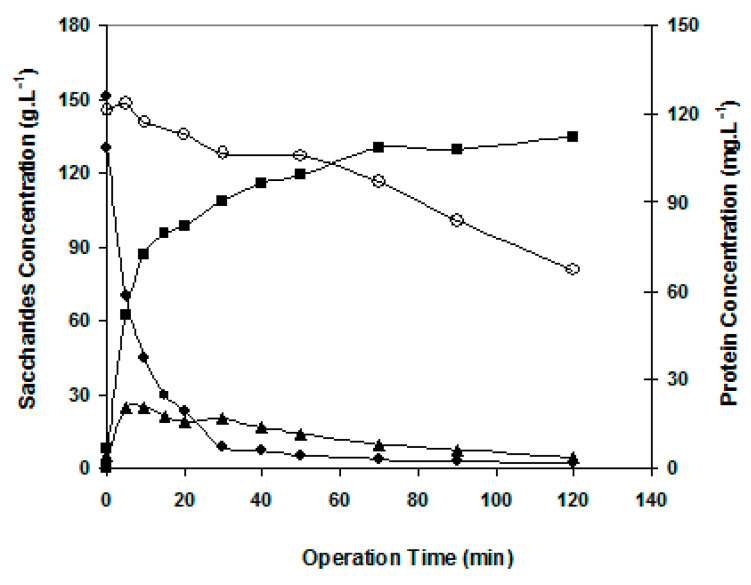
Time evolution of GOS production. Operating conditions: batch mode in Celfa unit without applied pressure, 1.7 m·s^−1^, 150 g·L^−1^ lactose, 10 U·mL^−1^ β-galactosidase, 40 °C and pH 6.5. Symbols: ■ monosaccharides, ♦lactose, ▲ GOS, ○ protein concentration (secondary axis).

**Figure 5:**
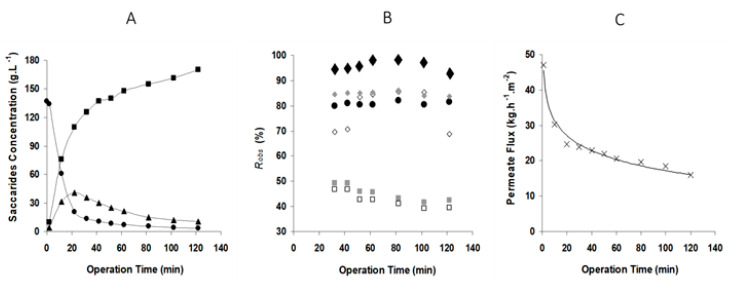
Time evolution of integrated GOS production and fractionation: (**A**) saccharide concentration profile, (**B**) saccharides rejection coefficients profile, (**C**) permeation flux profile. Operating conditions: batch mode in Celfa unit, 20 bar and 1.7 m·s^−1^, 150 g·L^−1^ lactose, 10 U·mL^−1^ β-galactosidase, 40 °C and pH 6.5. Symbols: ■ monosaccharides, ■ galactose, □ glucose, ● lactose, ▲ GOS, ♦ GOS-3, ♦ GOS-2′, ◊ GOS-2, × flux).

For comparison purposes, GOS production reactions were carried out in batch mode without permeation and at the same experimental crossflow conditions of the reaction carried out with simultaneous permeation, namely the crossflow velocity of 1.7 m·s^−1^. The reaction conditions were the same for the essays without and with simultaneous membrane permeation. The results in terms of component concentration profiles and fluxes are shown in Figure 4 and Figure 5.

Figure 4 shows data relative to lactose conversion in the situation of no permeation (no transmembrane pressure applied). Mono- and oligosaccharides were formed, and total GOS production reached a maximum of 25 g·L^−1^ observable at 10 min of reaction. Afterwards, the GOS synthesized were progressively hydrolyzed. The maximum GOS production occurred around 60% of lactose consumption. As reported by Mahoney (1998) [14], normally higher amounts of GOS are synthesized when more than 40% of initial lactose is hydrolyzed, because monosaccharide units are needed for this enzymatic synthesis.

The results for simultaneous bioconversion and separation by membrane filtration are presented in Figure 5, for the same crossflow velocity, but with an applied transmembrane pressure of 20 bar. The introduction of permeation leads to GOS production enhancement, as shown in Figure 5A. The maximum GOS production, 40 g·L^−1^, was observed around 20 min and was 64% higher in comparison with the same system without permeation. This increase in GOS production can be attributed to the partial removal of monosaccharides from the reactor during simultaneous reaction and permeation.

The analysis of the pattern of rejection to all saccharides during the filtration experiments (Figure 5B) indicates that, within 30 min, the crossflow filtration system was fairly stabilized and therefore the rejection coefficients remained constant until the end of the 120 min experiment. GOS-3 were almost totally retained, presenting 95 ± 3.0% of rejection; GOS-2′, GOS-2 and lactose presented 85 ± 0.8%, 76 ± 8.7% and 80 ± 2.6%, respectively and, on average, the disaccharides were retained at 80 ± 4.1%. Glucose and galactose were retained around 43 ± 3.0%. The rejection coefficients observed were much higher than those obtained for the reference solutes in binary solutions (Table 1).

Bouchoux et al. (2005) [15] investigated NF as a purification step, i.e., sugar removal, in the production process of lactic acid from sodium lactate fermentation broth. The authors observed that increasing complexity, i.e., from single-solute solutions to mixed-solute solutions, affected glucose retention, which was significantly lower in mixed-solute solutions, i.e., when sodium lactate is present. This decrease is such that the retentions of both solutes become comparable so that any purification is unachievable.

In this work, the presence of a solute of high molecular weight such as the enzyme (at 10 U·mL^−1^), in association with the high concentration of total sugars, 150 g·L^−1^, leads to complex feed solution structures that induce new membrane–solute–solvent interactions and, therefore, different selective mass transfer and different solute rejection coefficients.

De and Bhattacharya (1997) [16], who studied the UF of sucrose from a mixture of sucrose and poly(vinyl alcohol) (PVA) in a stirred cell, observed that the rejection is mostly governed by the gel layer of a higher MWCO membrane. The gel layer further rejects the solute in addition to the membrane, if it is partially retentive to the solute. For a 1-kDa membrane, rejection of sucrose was 86%, whereas true rejection by the membrane was 73% (in the absence of PVA). In the case of a 10 kDa membrane, rejection was 52%, whereas the membrane itself did not retain sucrose.

More recently, Fan et al. (2020) [12], also studying interactions between enzymatic reaction and multi-channel ceramic membrane filtration for the production of fructo-oligosaccharides using a 1-kDa UF membrane at ΔP of 0.5 bar (presumably the effective ΔP and not the applied pressure, taking into consideration the high total sugar content of 600 g/L) and 0.5 m·s^−1^ crossflow velocity, determined a stable permeation flux of ~7.5 L·h^−1^·m^2^ after an initial sharp decline. The flux of a pure enzyme solution (without the sugars) was 5.7-fold higher, and that was attributed to the ratio of viscosity of both solutions. Such a fact led these authors [12] to suggest the nonexistence of a significant interaction between the enzyme and the sugars that inhibits filtration. However, the sugars rejection profiles under simultaneous enzyme reaction and filtration were not presented.

In the present work, the high sugar concentration and the enzyme presence also had an important effect on the system permeation flux (Figure 5C). This flux declined fast in the first 20 min until reaching 25 kg·h^−1^·m^−2^ (the water flux is 370 kg·h^−1^·m^−2^ at 20 bar) and then decreased slowly until reaching 16 kg·h^−1^·m^−2^ after 2 h of filtration. Twenty bar is a very high pressure for an ultrafiltration operation and in fact is typical of nanofiltration. The high osmotic pressure associated with the high small sugar molecule concentration in the feed creates constraints to the process and the need for high applied pressure to counteract the osmotic effect and permit significant permeation fluxes.

The membranes prepared in this work are rated as tight UF membranes, due to their hydraulic permeability of 15 ± 3 Kg·h^−1^·m^−2^·bar^−1^ (Figure 3) and partial rejection characteristics towards divalent ions and trisacharide raffinose (R_obs_ = 25%; Table 1), which already reveals a trend of NF behavior. The estimation of the MWCO was not performed in this work. However, in an NF study used to fractionate an enzyme-free GOS mixture at a total sugar content of 150 g/L the osmotic pressure differences (Δπ) ranged from 7.5 bar to 13.5 bar for applied pressures in the range of 10–25 bar, varying with the sugars’ rejection performances (Table 3 of Ref. [2]). The saccharide rejection behaviors were similar to the ones found in the present work under simultaneous enzyme reaction and membrane filtration. This justifies the need for higher applied pressures to counteract osmotic pressure of this magnitude. It is worth mentioning that, in the present conditions, viscosity effects should not be relevant for the flux behaviors since a lactose solution at 15% (*w*/*w*) and 40 °C has a viscosity of just 1.03 mPa·s [17].

**Figure 6:**
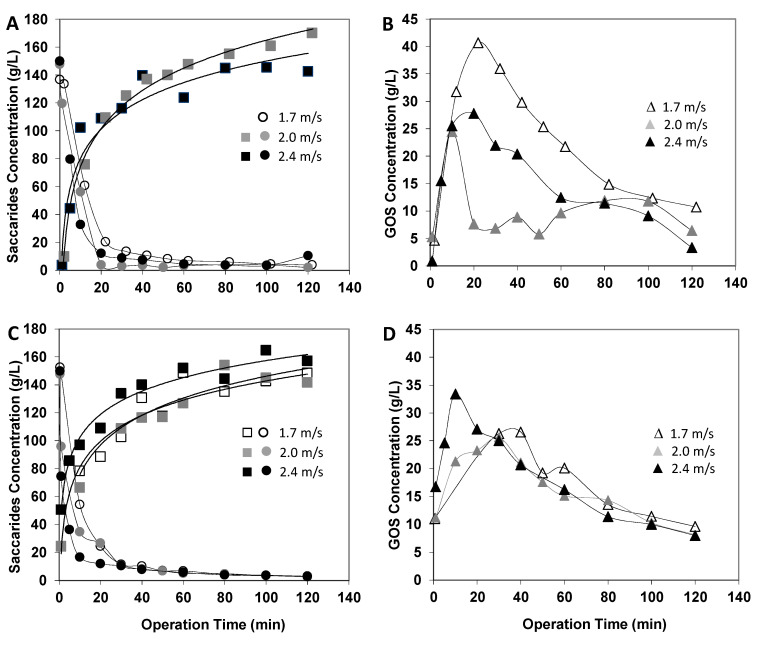
Effect of the fractionation operating parameters on the enzymatic reaction outcome: transmembrane pressure of 20 bar (**A**,**B**) and of 24 bar (**C**,**D**) and crossflow velocities ranging from 1.7 to 2.4 m·s^−1^. Reaction conditions as reported in Figure 1. Symbols: lactose (circles, ● ● ○), monosaccharides (squares, ■ ■ □), total GOS (triangles, ▲ ▲ Δ).

**Figure 7:**
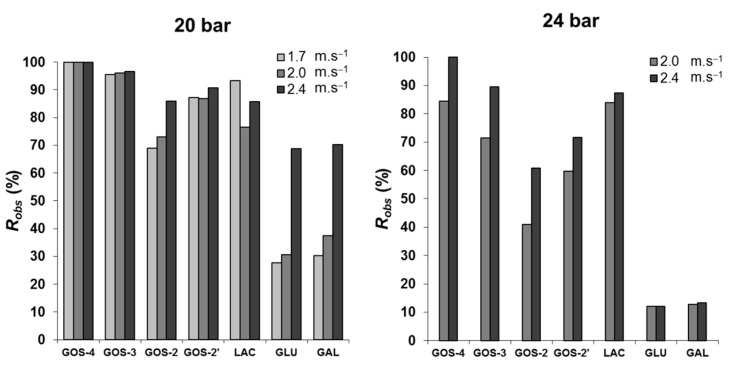
Rejection coefficients observed at 120 min of GOS synthesis and fractionation at 150 g·L^−1^ initial lactose concentration, 40 °C, pH 6.5, at 20 bar (**left graph**) and 24 bar (**right graph**), using different crossflow velocities.

The effects of the variation of fractionation operating parameters, namely crossflow velocity and transmembrane pressure, on GOS synthesis and on solutes’ rejection pattern are presented in Figure 6 and Figure 7, respectively.

After a drastic decline, approximately at 20 min of reaction, there was practically no difference in the lactose concentrations, no matter the operating conditions (Figure 6A,C). Regarding monosaccharides, the pattern of concentration variation with time was similar for all experimental conditions. Nevertheless, the pressure increase from 20 bar to 24 bar resulted in a different influence of the crossflow velocity parameter on the final monosaccharide concentration. Very different patterns were displayed for GOS concentration variation with time. After a steep increase to a GOS concentration maximum, there was a smooth decay over time. The GOS synthesis/hydrolysis evolution is strongly dependent on crossflow velocity for the lower pressure and becomes fairly independent at the higher transmembrane pressure of 24 bar, as seen in Figure 6B,D, respectively. For the lower pressure, when the velocity decreased the total concentration of GOS reached higher values, and the time needed to reach this maximum increased. At the lowest pressure and lowest crossflow velocity of 1.7 m·s^−1^, there was a maximal GOS concentration of 40 g·L^−1^, whereas at the higher pressure of 24 bar, the GOS concentration only reached a maximum value of 33 g·L^−1^, and that was obtained at the maximum velocity of 2.4 m·s^−1^.

Figure 7 shows the crossflow velocity effect on the *R_obs_*, at 120 min of filtration, for the transmembrane pressures of 20 bar (left) and 24 bar (right). The increase in the velocity did not lead to variations of great magnitude in *R_obs_*, with exception for monosaccharides at 20 bar. In fact, when shifting from the lowest to the highest crossflow velocity, from 1.7 to 2.4 m·s^−1^, a change from 28% to 70% rejection was induced, while at 24 bar they permeated much more easily, and the low *R_obs_* (12.5%) became velocity-independent. Regarding trisaccharides, GOS-3 were almost totally retained at 20 bar for all velocities. They were partially retained at the higher operating pressure of 24 bar, and its rejection coefficient became dependent on crossflow velocity—*R_obs_* of 70% at 2.0 m·s^−1^ and almost 90% at 2.4 m·s^−1^. The rejection to lactose and the other two disaccharide fractions, GOS-2 and GOS-2′, were affected by changes to both applied pressure and crossflow velocity. Particularly for the higher pressure of 24 bar, at 2.0 m·s^−1^, the *R_obs_* increased in the order of GOS-2 < GOS-2′ < lactose from about 40 to 60% and to 80%. Furthermore, at the higher velocity of 2.4 m·s^−1^, the rejection coefficients for each one of these fractions increased.

Low values of monosaccharide retention play a major role in the shift of the enzymatic hydrolysis–synthesis equilibrium and, in addition, the removal of monosaccharides from the retentate is very interesting from the technological point of view. Even so, the operating conditions applied should not lead to increased permeation of GOS, as it would compromise the production/recovery yield. A transmembrane pressure of 24 bar is less favorable for this process objective.

Additionally, from the above discussion of Figure 7, it seems that the reasons for the different profiles of GOS concentration (Figure 6B,D) are not only related with differences of saccharide rejections through the membrane but most likely pressure and shear effects on the β-galactosidase activities.

**Figure 8:**
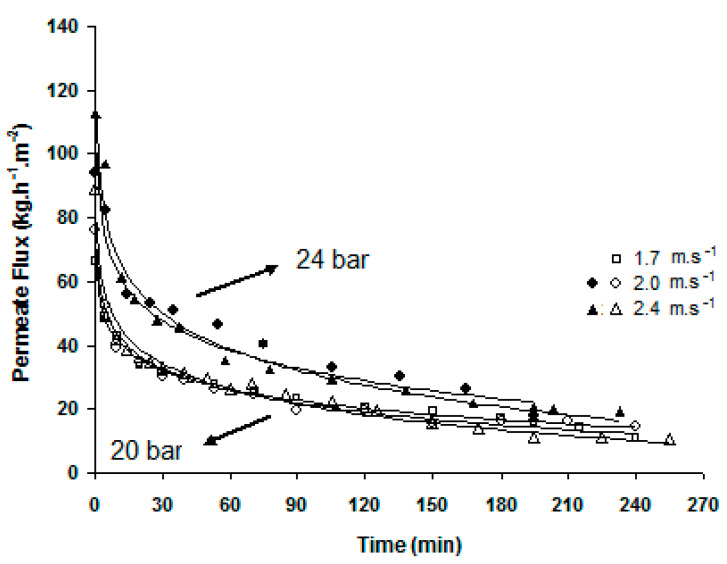
Effects of pressure and crossflow velocity on the permeation fluxes of the membrane reactor.

Figure 8 shows the crossflow velocity effect on the permeation flux. The pressure increase led to an increase in both initial and final flux values, although practically no permeate flux improvement was observed with the crossflow velocity increase. At 20 bar, for the three velocities tested the average permeate flux was exactly the same: 25 kg·h^−1^·m^−2^. At 24 bar, the average permeate fluxes were very similar as well: 39 kg·h^−1^·m^−2^ at 2.0 m·s^−1^ and 34 kg·h^−1^·m^−2^ at 2.4 m·s^−1^ crossflow velocity. An important and constant reduction in the initial permeation flux occurred; a reduction of 80% on average, for the first 60 min, indicates that the development of the concentration polarization layer was occurring, which can further lead to membrane fouling and namely to colloidal fouling [18].

De and Bhattacharya (1997) [16] observed that flux declined as a gel layer of PVA grew with time and that the steady-state flux value was the same as that of a PVA solution alone (in the absence of sucrose). In opposition to observations in the present work, the former authors stated that the steady-state flux depended on the hydrodynamic conditions but was independent of pressure. However, one must consider that the range of process transmembrane pressures, crossflow velocities and type and concentration of polymers used in both works are different.

Table 2 presents the composition in the feed tank after 120 min of filtration. The monosaccharides were the main component, representing almost 90% of the total composition in the feed tank and reinforcing the great hydrolytic activity. Traces of GOS-4 were observed for all tested operating conditions. The small amounts of lactose indicate that all the substrate was consumed, and some of the GOS previously synthesized had been hydrolyzed (maximum production around 20–30 min). The reactor was operating in a discontinuous mode, as an attempt to understand a complex reaction–permeation system. If the same reaction is carried out in continuous mode, the residence time should be chosen according to the results of the maximal GOS production.

### 3.2. Membrane Regeneration

As seen before in Figure 8, the permeation flux had a similar evolution with time for the transmembrane pressures of 20 bar and 24 bar and, for both pressures, was independent of the crossflow velocity, leading to the conclusion that the hydrodynamic conditions of feed circulation were optimal. Nevertheless, initial (instant) fluxes may be sufficiently different to induce a dissimilar distribution of the partial contributions to the overall resistance [19].

**Figure 9:**
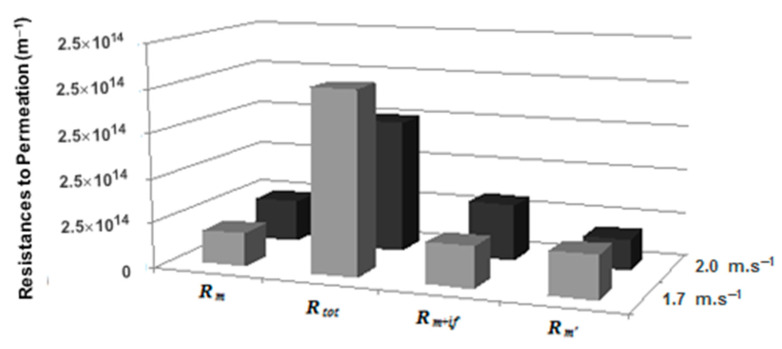
Overall resistances to permeation: *R_m_*—membrane resistance, *R_tot_* and *R_m+if_*—total and membrane with irreversible fouling resistances, respectively, *R_m′_*—membrane resistance, after reaction/separation test and regeneration. Transmembrane pressure of 20 bar and crossflow velocities of 1.7 and 2.0 m·s^−1^.

Accordingly to definitions depicted in Section 2.5, Figure 9 shows the resistances to permeation at 20 bar for 60 min, using distinct crossflow velocities of 1.7 m·s^−^^1^ and 2.0 m·s^−^^1^. In these test conditions, the total resistance to permeation was 1.8 × 10^14^ m^−^^1^ and 1.4 × 10^14^ m^−^^1^, respectively for 1.7 m·s^−^^1^ and 2.0 m·s^−^^1^.

The high shear tangential to the membrane surface sweeps deposited particles away; thus, the fouling layer on the surface of the membrane should be reduced. Actually, it can be observed that the main contribution to the *R_tot_* is conferred by the concentration polarization layer plus that of reversible fouling, which increases when the crossflow velocity decreases.

Despite the intense research on membrane fouling, namely on membrane bioreactors [20], many questions still remain unanswered. In the light of the complexity of the present system, one can conclude that an 18% increase in crossflow velocity is sufficient to significantly reduce the total resistance to permeation (Figure 9). By subtracting the *R_m+if_* resistance term from the total resistance, the concentration polarization and reversible fouling contributions emerge as the major ones. Furthermore, looking at the other calculated resistances and comparing them with that of a clean membrane, one tends to agree that it is rather easy to regenerate the membrane.

## 4. Conclusions

In this work batch GOS synthesis reactions with soluble enzymes are processed simultaneously with crossflow ultrafiltration in a P-28 Celfa unit (400 mL working volume for a membrane filtration area of 26.4 cm^2^). The GOS syntheses were carried out at 40 °C, using 150 g·L^−1^ lactose and 10 U·mL^−1^ β-galactosidase under filtration conditions of 1.7, 2.0 and 2.4 m·s^−1^ crossflow velocity and applied relative pressures of 20 and 24 bar. Permeation flux, enzyme substrate and product molecule (namely GOS) profiles during reaction and rejections by the cellulose acetate membrane were investigated. The present work demonstrates that selected and controlled pressure and shear conditions in an enzyme membrane bioreactor system under simultaneous ultrafiltration permeation leads to favorable dislocation of the β-galactosidase activity towards optimal GOS production. Synthesis of GOS in membrane bioreactors is feasible and advantageous. Under the best crossflow filtration conditions studied herein, an applied ΔP of 20 bar and 1.7 m·s^−1^ and a tight cellulose acetate membrane that shows NF character under operation, the amount of GOS produced was 64% higher in comparison to the reaction carried out in the same enzymatic fresh mixture used without permeation.

The presence of a solute of high molecular weight such as the enzyme, in association with the high concentration of total sugars, led to complex feed solution structures, that induced new membrane–solute–solvent interactions and, therefore, different selective mass transfer and different solute rejection coefficients, when compared to data obtained in binary solutions at a very low solute concentration.

During simultaneous synthesis and permeation, there were no crossflow velocity effects on permeate flux, which resulted in average values of 25 kg·h^−1^·m^−2^ for an applied ΔP of 20 bar. The flux could only be increased with a pressure increase (up to 39 kg·h^−1^·m^−2^ at 24 bar) and at the expense of reduced retention of the enzymatically formed galacto-trisaccharides.

The tight ultrafiltration membrane installed displayed total permeation of mono- and disaccharides and a rejection coefficient of 25% to trisaccharides for single-solute aqueous solutions. However, during simultaneous synthesis and fractionation at 20 bar, GOS-3 were totally retained, and regardless of the flow speed, GOS-2 and monosaccharides were retained at ~80% and ~40%, respectively (values dependent on crossflow velocity). This membrane behavior under operation is consistent with an NF behavior. The membrane rejection profiles were affected by operational conditions, and mono- and disaccharide rejections were the most affected ones. Particularly, GOS-3 rejection decreased with an ΔP increase (24 bar), to values of 70% and 90%, at 1.7 m·s^−1^ and 2.4 m·s^−1^, which is not adequate for the designed process.

The GOS synthesis/hydrolysis evolution was dependent on crossflow velocity for the lower pressure and became fairly independent at the higher transmembrane pressure of 24 bar.

The present work reinforces the promise of the integration of membrane separation and reaction processes and provides preliminary data for selecting adequate residence time to operate a continuous membrane bioreactor that maximizes GOS production (with retention of the enzyme and oligosaccharides).

## Figures and Tables

**Table 1:** Reference solutes rejection coefficients of CA membranes at 25 °C (Adapted from [2]).

Reference Solutes	NaCl	Na_2_HPO_4_	Glucose	Lactose	Raffinose
Rejection (%)	3	25	8	3	25

**Table 2:** Feed tank composition at 120 min of GOS synthesis and fractionation at 150 g·L^−1^ initial lactose concentration, 40 °C, pH 6.5, at 20 and 24 bar, with different crossflow velocities.

Pressure	Velocity	Concentration (g·L^−1^)					
(bar)	(m.s^−1^)	LAC	GOS-4	GOS-3	GOS-2_t_^¥^	GLU	GAL
	1.7	7.6	0.3	1.8	4.5	89.2	90.9
20	2.0	10.3	0.4	1.9	4.7	79.6	85.7
	2.4	5.6	0.5	3.9	10.5	94.4	96.7
24	**2.0**	12.0	0.6	3.0	6.3	90.9	91.9
	2.4	9.5	0.7	3.3	6.6	93.3	94.3

^¥^ GOS-2_t_ = GOS-2 + GOS-2′.
